# Unexpected severe malaria in a postoperative patient, New York, USA

**DOI:** 10.1186/s12879-024-09272-6

**Published:** 2024-04-15

**Authors:** Alan Bulbin, Julia Shen, Carol Liotta-Bono, Tahir Ahmad

**Affiliations:** https://ror.org/00mj4n083 grid.416387.f 0000 0004 0439 8263 St. Francis Hospital and Heart Center, 100 Port Washington Blvd, 11576 Roslyn, NY USA

## Abstract

Severe malaria is not routinely considered when evaluating a febrile patient in the postoperative setting. Common bacterial infections, along with adverse drug reactions, are the usual differential concerns. We present a case of severe malaria emerging unexpectedly eight days after routine craniotomy.

## Background

When encountered within the first 48 hours, post-surgical fever is usually from non-infectious causes. Beyond this, the differential diagnosis includes bacterial infections such as surgical site/wound, urinary tract, and pneumonia. The possibility of hypersensitivity reactions to newly introduced drugs should also be considered [1]. Malaria has been reported in the postoperative setting, but typically only from endemic regions or returned travelers [2, 3]. Malaria has rarely been acquired via transfusion following open heart surgery [4]. However, case reports of autochthonous malaria emerging postoperatively are lacking.

## Case Presentation

SC is a 65 year old male with prior history of hypertension and hyperlipidemia, maintained on long term aspirin therapy, who suffered a mechanical fall with head trauma on September 15, 2023. He presented to a New York City hospital, was found to have a left subdural hematoma, leading to left craniotomy, and surgical evacuation on September 18th. He transferred to our institution on September 21st for postoperative management. His subsequent hospital course was without incident. He recovered well from his surgery, remained fever free; CBC and liver enzymes all were normal. He did not require a blood transfusion. Metformin was initiated for newly diagnosed diabetes, along with levetiracetam for seizure prophylaxis, while maintained on losartan, metoprolol, and atorvastatin. He was discharged home on September 24th.

The patient returned to our Emergency Department (ED) on October 1st with fever, malaise, and dark urine progressing over four days. On presentation, vital signs were as follows: temperature 100.8 °F, pulse 92 beats/min, blood pressure 98/62 mm Hg, and oxygen saturation 100% on room air. He was alert and oriented. The physical exam otherwise was unremarkable; the left craniotomy incision was clean and dry; skin demonstrated no rashes or lesions. Laboratory results demonstrated elevated lactic acid and liver enzymes, mild hyponatremia and thrombocytopenia. White blood cell count and hemoglobin values were normal. No eosinophilia was noted (Table 1). Computerized Tomography (CT) imaging of head, chest, abdomen, pelvis and Magnetic Resonance Imaging (MRI) of brain did not reveal any acute process.

Initial treatment in the ED included IV fluids, antipyretics and antibiotics (vancomycin and piperacillin-tazobactam). Neurosurgery assessment determined no clinical signs of meningitis, brain abscess, cerebral edema or infection from his recent cranial surgery. The subdural hematoma was improved.

Over the next day, the patient experienced high fevers (peak of 104 °F) as well as progressive anemia, thrombocytopenia, and an uptrend in bilirubin (Table 1). Hematology was consulted; the peripheral blood smear was reviewed, revealing many ring form intraerythrocytic organisms, concerning for babesiosis vs. malaria.

**Fig. 1:**
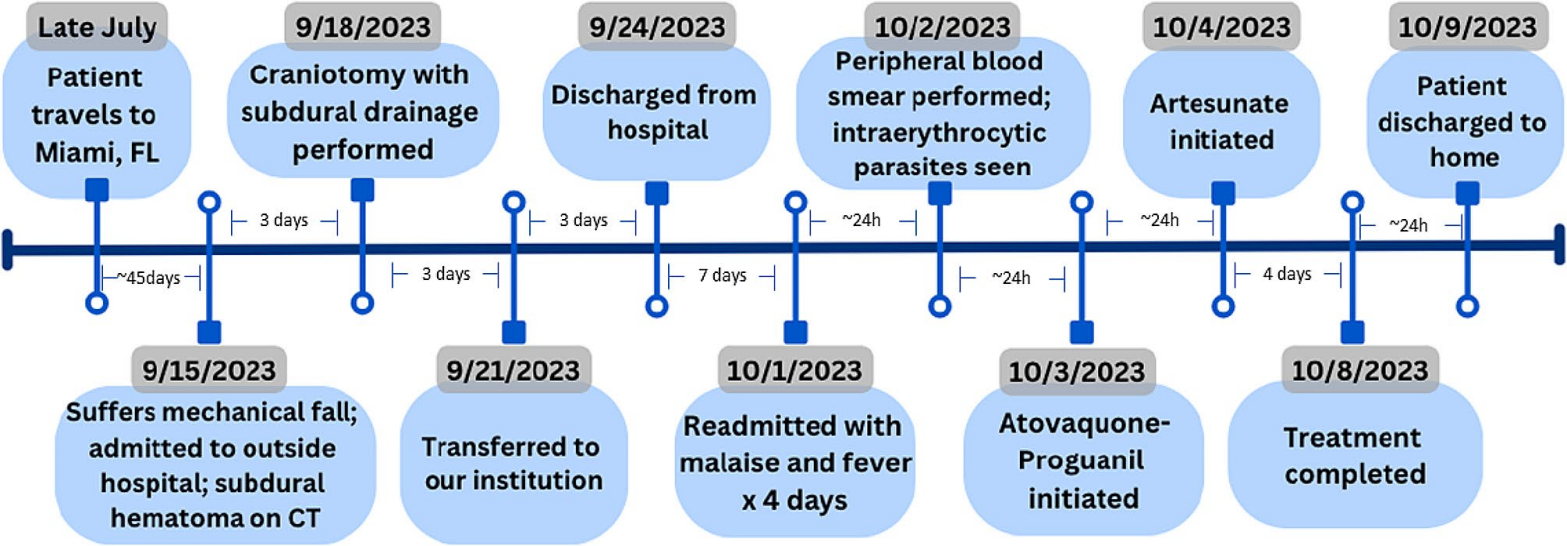
Timeline of Events and Sequence of Therapy

The patient lives in a developed area of suburban Yonkers, New York. The nearest wooded area is two miles from his home, where deer have been seen, although he denied any known tick bites. His only travel was to Miami Beach and the Florida Keys eight weeks prior to his brain injury (Fig. 1). He works as a food vendor, selling fruits and vegetables at a large market in Hunts Point, New York. He reported mosquito bites both at home and work. He was working up to September 15th, when he fell at home. He has no history of substance use or needle sharing. He has no history of prior malaria.

Based on the report from Hematology, with guidance from Infectious Disease, antibiotic coverage was switched to azithromycin 500 mg daily, doxycycline 100 mg twice daily, and atovaquone 750 mg twice daily on October 2nd.

A formal thick and thin blood smear was sent to our reference lab on October 2nd. The next day, the report confirmed *Plasmodium falciparum* detected with > 15% parasitemia. A diagnosis of severe malaria was made, and available atovaquone-proguanil (Malarone™) 250 − 100 mg (4 tablets) daily was initiated. Intravenous Artesunate was ordered via the commercial supplier, which arrived on October 4th, and was immediately started. The patient completed two full doses of atovaquone-proguanil while awaiting the arrival of Artesunate.

Artesunate was initiated at a dose of 2.4 mg/kg/dose (180 mg) IV every 12 h x 3 doses at 0, 12 and 24 h over October 4th and October 5th. On October 6th, Artesunate 180 mg IV daily was continued. A repeat blood smear from October 5th, after two doses of both atovaquone-proguanil and Artesunate, revealed a significant decrease in parasitemia to 0.3%.

By day 3 of therapy (October 6th ), the patient’s fever curve improved, total bilirubin had normalized with AST, ALT and LDH slowly declining (Table 1). Thrombocytopenia was improving as well, and the patient felt better overall. Repeat blood smear on October 6th showed further improvement in parasitemia to 0.1%. Due to these improvements, the decision was made to limit Artesunate to one more daily dose for a total of 4 days and convert the patient back to atovaquone-proguanil to complete the oral course. Artesunate was stopped on October 7th, and the last dose of atovaquone-proguanil was administered October 8th (Fig. 1). A final blood smear on October 8 showed no intraerythrocytic parasites.

There were no immediate adverse events related to malaria treatment. The patient tolerated therapy well and was discharged home on October 9th. On the day of discharge, the patient’s labs continued to normalize (Table 1).

**Table 1:** Patient’s Laboratory Data Summary

	10/1	10/2	10/3	10/4	10/5	10/6	10/7	10/8	10/9
**AST**	91	447	259	155	236	196	119	100	62
**ALT**	71	187	165	133	127	118	119	114	98
**T Bili**	1.7	6.1	2.6	2.5	1.9	1.1	0.9	1.0	1.0
**LDH**	-	1079	965	-	1577	1331	958	716	590
**PLT**	133	56	46	63	66	82	81	80	93
**H/H**	15.5/43.8	12.4/34.6	11.8/32	9.1/24.9	9.9/27.4	9.3/25.5	9.6/26.4	9.3/25.2	9.7/26.6
**Malaria** **Blood Parasite**		PLASMODIUM FALCIPARUM > 15.0%			PLASMODIUM FALCIPARUM 0.3%	PLASMODIUM FALCIPARUM < 0.1%		Negative	

## Discussion

This case of *P. falciparum* malaria was completely unexpected. The patient developed fever eight days after surgery without any suspicious travel history. Initial concerns of a routine postoperative bacterial infection were promptly excluded. The possibility of a drug reaction from levetiracetam was considered in view of liver injury, but no rash or eosinophilia was seen. Ultimately, the high fever, typical progression of laboratory parameters, including evidence of hemolysis, and intraerythrocytic parasites seen on the peripheral blood smear led to the diagnosis of malaria, as later confirmed on formal thick and thin smear.

The initial peripheral smear finding on October 2nd was the first alert to a possible bloodborne pathogen. However, malaria was still difficult to consider, especially in a patient with no relevant travel history. Locally acquired Babesiosis is more commonly encountered in our region/experience. Due to low volume, our centralized microbiology laboratory does not perform formal malaria smears, opting to send specimens to an offsite reference lab (Quest Diagnostics®).

As of October 3rd, the malaria diagnosis was confirmed and reported to the New York State Department of Health (NYSDOH) as required. However, the source of infection was difficult to determine. The patient had been hospitalized, and upon discharge, did not leave his house during the two weeks prior to his febrile presentation on October 1st.

Cases of malaria locally acquired in Florida have been reported [5], but his travel to South Florida was eight weeks prior. Moreover, the cases reported in Florida were all from *Plasmodium vivax* and identified only in Sarasota County [5]. The incubation period for the different forms of malaria can be variable, but *P. falciparum* (as diagnosed in our patient) is typically associated with shorter incubation times of 7–20 days [6]. His work selling international fruits and vegetables raised the possibility of “baggage” malaria, meaning presumed occupational exposure from an infectious mosquito imported via the shipped produce directly. Otherwise, local exposure from a New York resident anopheles mosquito vector should be considered. Finally, the possibility of nosocomial malaria could be questioned. Cases of hospital-acquired malaria with breaks in standard precautions have been reported [7, 8]. These reports highlight the possible link between a non-traveler and a malaria-infected returned traveler, if hospitalized together. This is even more relevant if genomic typing between the two match [7, 8]. 

The Center for Disease Control and Prevention (CDC) malaria treatment guidelines include prompt and aggressive treatment with parenteral Artesunate for patients with manifestations of severe malaria [9]. Severe malaria symptoms may include impaired consciousness/coma, hemoglobin < 7 g/dL, acute kidney injury, acute respiratory distress syndrome, circulatory collapse/shock, acidosis, jaundice, disseminated intravascular coagulation, and/or parasite density of ≥ 5% [9]. Our patient initially was found to have > 15% parasite density. If Artesunate is not immediately available, treatment with any other active agent should be initiated.

Our patient received doxycycline, atovaquone, and then combination atovaquone-proguanil while awaiting the arrival of Artesunate.

Artesunate is an intravenous antimalarial agent that is metabolized to dihydroartemisinin (DHA) [10]. Artesunate and DHA are activated by heme iron binding, resulting in oxidative stress, inhibition of protein and nucleic acid synthesis, and a decrease in parasite growth and survival [10]. It is dosed at 2.4 mg/kg/dose at 0 h, 12 h, and 24 h, followed by once daily for up to a total of 7 days depending on patient response [9]. After intravenous therapy is completed, it is recommended to complete a full course with an oral regimen [9]. Artesunate is generally well tolerated, but can cause hypersensitivity reactions (hypotension, anaphylaxis, dyspnea, urticaria, and rash) during administration, as well as delayed hemolytic anemia after treatment [10]. All persons treated for severe malaria with intravenous Artesunate should be monitored weekly for up to four weeks after treatment initiation for evidence of hemolytic anemia [9]. 

## Conclusion

Despite being completely unexpected, with no typical travel history, the clinical and laboratory parameters ultimately led to the diagnosis of severe *P. falciparum* malaria in our postoperative patient. Artesunate was ordered and delivered via the manufacturer within less than 24 hours. The patient was discharged after a satisfactory clinical response with clearance of parasitemia. He remains relapse free with no evidence of hemolytic anemia.

We believe this is a case of locally acquired malaria (autochthonous) as outlined above. In these situations, it is vital NYSDOH and CDC investigate and discover the source of infection to detect a possible malaria outbreak. The investigation is ongoing. They report a possible nosocomial exposure involving a returned traveler at the outside hospital. As of February 22, 2024, our last communication with NYSDOH Bureau of Communicable Disease Control, there have been no additional community-associated or work-related malaria cases identified.

## Data Availability

Relevant patient-specific data included in manuscript. No additional raw data.
